# Exogenous Polyamines Only Indirectly Induce Stress Tolerance in Wheat Growing in Hydroponic Culture under Polyethylene Glycol-Induced Osmotic Stress

**DOI:** 10.3390/life10080151

**Published:** 2020-08-14

**Authors:** Izabela Marcińska, Kinga Dziurka, Piotr Waligórski, Franciszek Janowiak, Edyta Skrzypek, Marzena Warchoł, Katarzyna Juzoń, Kamila Kapłoniak, Ilona Mieczysława Czyczyło-Mysza

**Affiliations:** The Franciszek Górski Institute of Plant Physiology, Polish Academy of Sciences, Niezapominajek 21, 30-239 Kraków, Poland; i.marcinska@ifr-pan.edu.pl (I.M.); k.dziurka@ifr-pan.edu.pl (K.D.); p.waligorski@ifr-pan.edu.pl (P.W.); f.janowiak@ifr-pan.edu.pl (F.J.); e.skrzypek@ifr-pan.edu.pl (E.S.); m.warchol@ifr-pan.edu.pl (M.W.); k.juzon@ifr-pan.edu.pl (K.J.); k.kaploniak@ifr-pan.edu.pl (K.K.)

**Keywords:** osmotic stress, PEG, polyamines, fluorescence kinetics, biochemical factors, yield, *Triticum aestivum* L.

## Abstract

The aim of the present study was to evaluate the effect of osmotic stress caused by polyethylene glycol (PEG) 6000 in hydroponic culture on wheat seedlings of drought-resistant Chinese Spring (CS) and drought-susceptible SQ1 cultivar, and to examine the alleviative role of exogenous polyamines (PAs) applied to the medium. The assessment was based on physiological (chlorophyll *a* fluorescence kinetics, chlorophyll and water content) as well as biochemical (content of carbohydrates, phenols, proline, salicylic and abscisic acid, activity of low molecular weight antioxidants) parameters, measured after supplementation with PAs (putrescine, spermidine and spermine) on the 3rd, 5th and 7th day of the treatment. The results indicate that PAs ameliorate the effects of stress, indirectly and conditionally inducing stress tolerance of wheat seedlings. In contrast to the susceptible SQ1, the resistant CS cultivar activated its protective mechanisms, adjusting the degree of their activation to the level of the stress, depending on the genetic resources of the plant. Increased accumulation of antioxidants in the resistant CS in response to stress after the application of PAs confirms the hypothesis that PAs are involved in the signaling pathway determining the antioxidative response and the tolerance of wheat plants to drought stress.

## 1. Introduction

**Figure 1:**
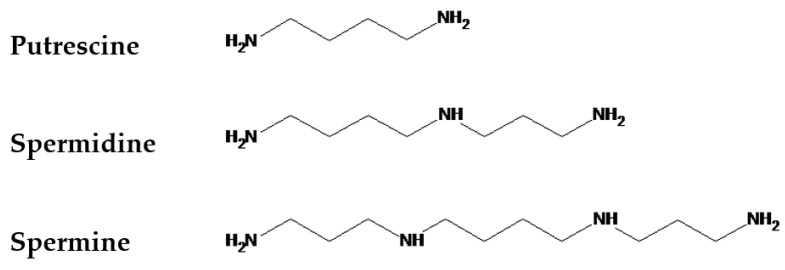
The structure of polyamines.

Polyamines (PAs) are polycationic compounds of low molecular weight, which have been proposed as a new category of plant growth regulators involved in a large spectrum of physiological processes; moreover, they have been shown to be an integral part of plant stress response [1,2,3,4]. The most common PAs in living organisms are putrescine, spermidine and spermine [5]. These are aliphatic compounds, among which the simplest—putrescine (Put)—is a diamine, spermidine (Spd)—a triamine, and spermine (Spm)—a tetraamine (Figure 1).

The presence of several positively charged amino groups in PA molecules gives them highly polar properties, thanks to which they interact strongly with dipole molecules of water. These properties give PAs excellent water solubility and the ability to bind a large number of water molecules by forming a solvation sheath [6]. PAs are able to bind with other biologically important molecules containing anionic groups, including phospholipids which are a part of biological membranes [7], and proteins with various functions, such as membrane and receptor proteins, ion pumps, enzymes and structural proteins [8]. Biological structures with which PAs are combined increase their mechanical stability. Low molecular weight and excellent water solubility result in high mobility of PA molecules which facilitates their transport and allows them to act as regulators of physiological functions [9]. PAs regulate the activity of many types of ion channels [10] and also participate in the regulation of programmed cell death processe [11]. Moreover, PAs are a pool of substances involved in the mechanism of intracellular nitrogen to carbon balance [12]. Since PAs contain a lot of nitrogen, some authors have hypothesized that they could form a source of nitrogen for the synthesis of amino acids, and in the case of plants also chlorophyll [13]. At the cellular level, PA concentrations in plants play a role in many biochemical processes which require maintaining a specific homeostasis [11]. 

The polycationic nature of PAs means that these compounds can prevent water loss under osmotic stress because an increase in their content in the solution significantly increases the kinetic energy required for the water molecule to enter the gas phase. The phenomenon of PAs accumulation in plants was described by Maiale et al. [14] during exposure to drought, but a great variety of reactions were observed, depending on the species, organ, and in particular, on the time scale. In cereal plants, exposure to various environmental factors causes a higher increase particularly in the content of putrescine in comparison to other PAs [15]. Many authors have described the alleviating effects of PAs on the detrimental impact of environmental stress in various plant species. For example, putrescine prevents the damage of photosynthetic apparatus [16,17,18] and some DNA damage [19]. Under osmotic stress, a stabilizing effect of PAs on thylakoid membranes was also observed [20]. The degree of PAs accumulation is determined by the starting point and duration of the stress [14,21]. Due to the postulated participation of PAs in drought response processes, the interaction between abscisic acid (ABA) and PAs is particularly interesting. Liu et al. [22] reported an increase in ABA concentration in wheat seeds treated with PAs, among which putrescine was found to have the most stimulating effect. In general, the physiological effects of PAs rely on interactions with other compounds present in the cell, including carbohydrates, proline, phenols, as well as other low molecular weight antioxidants, salicylic and abscisic acid.

The aim of the present work was to evaluate the effect of osmotic stress caused by PEG 6000 in hydroponic culture on wheat seedlings, and to examine the alleviative role of PAs applied to the medium through physiological as well as biochemical analyses. The main objective was to verify the hypothesis that PAs are able to increase water deficit tolerance and ameliorate the harmful impact of osmotic stress in wheat plants. 

## 2. Results

### 2.1. Physiological Parameters

Osmotic stress was found to affect chlorophyll fluorescence parameters, water status, water soluble carbohydrates, phenols, ABA, SA and proline content in drought resistant and drought susceptible wheat cultivars. In this study, we evaluated the ameliorative influence of the supplementation of PEG solution in hydroponics culture with polyamines—Put, Spd and Spm—in CS and SQ1 wheat cultivars. We examined the mechanisms of osmotic stress amelioration by observing selected physiological and biochemical characteristics, as well as water relations in seedling leaves and yield.

**Figure 2:**
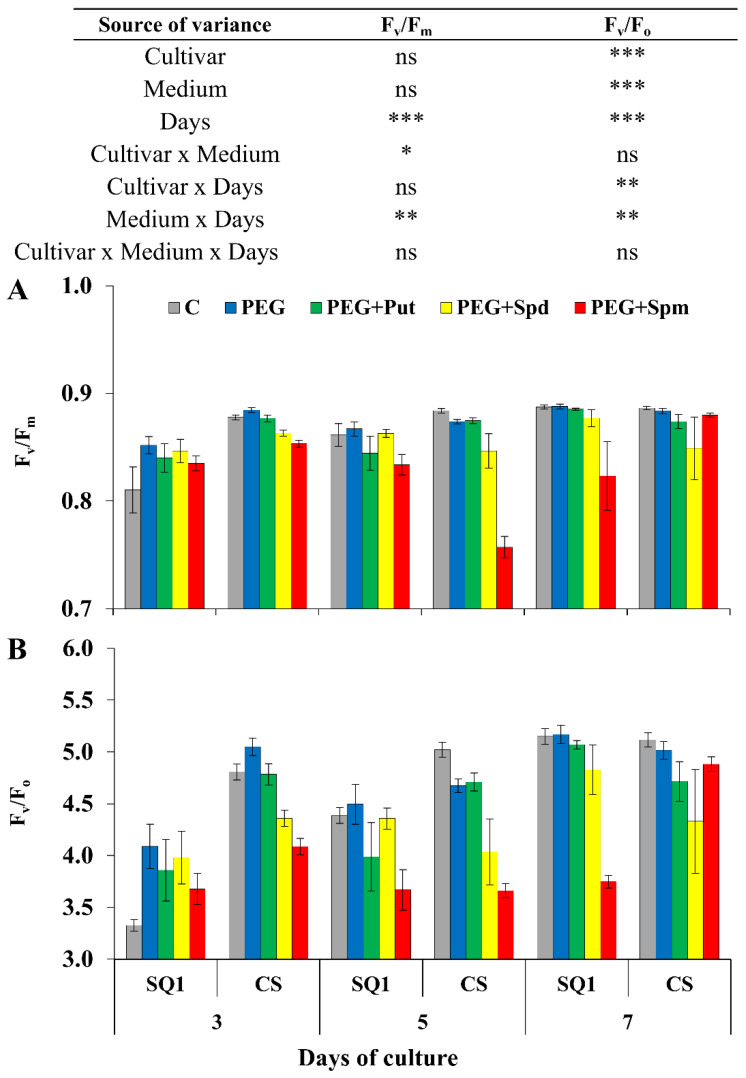
Chlorophyll *a* fluorescence parameters F_v_/F_m_ (**A**) and F_v_/F_o_ (**B**) in leaves of two wheat cultivars—CS and SQ1—on the 3rd, 5th and 7th day of PEG-induced osmotic stress and supplementation with PAs, resulting in a total of five treatments: Control, PEG, PEG+Put, PEG+Spd and PEG + Spm. The results of multiple factor analysis of variance (ANOVA) are presented in the table above the chart. The sources of variance of the measured parameters were as follows: two cultivars, five media, three measurement days, and their interactions. *, **, ***, significant at *p* ≤ 0.05, 0.01, 0.001, respectively; ns, not significant. Each bar represents a mean of 6 replicates ± SE.

Chlorophyll *a* fluorescence (CF) kinetics provided prompt quantitative information on the response of the photosynthetic apparatus to environmental changes. After drought treatment F_v_/F_m_ parameter differed significantly depending on the measurement day and the interaction of medium and day, though it was visibly reduced only in the presence of spermine on the 5th day for CS and 7th day for SQ1 (Figure 2A). The values of this parameter were similar at the other measurement points during 7 days of stress. Higher significant differences were observed for F_v_/F_o_ depending on the cultivar, medium and treatment day (Figure 2B). On the 3rd day PEG caused a ca. 8% increase in F_v_/F_o_ compared to the control. Changes of this parameter were similar for control, PEG and supplementation with putrescine. Compared to these media, in the presence of spermidine and spermine, a decrease in F_v_/F_o_ was observed for both cultivars.

**Figure 3:**
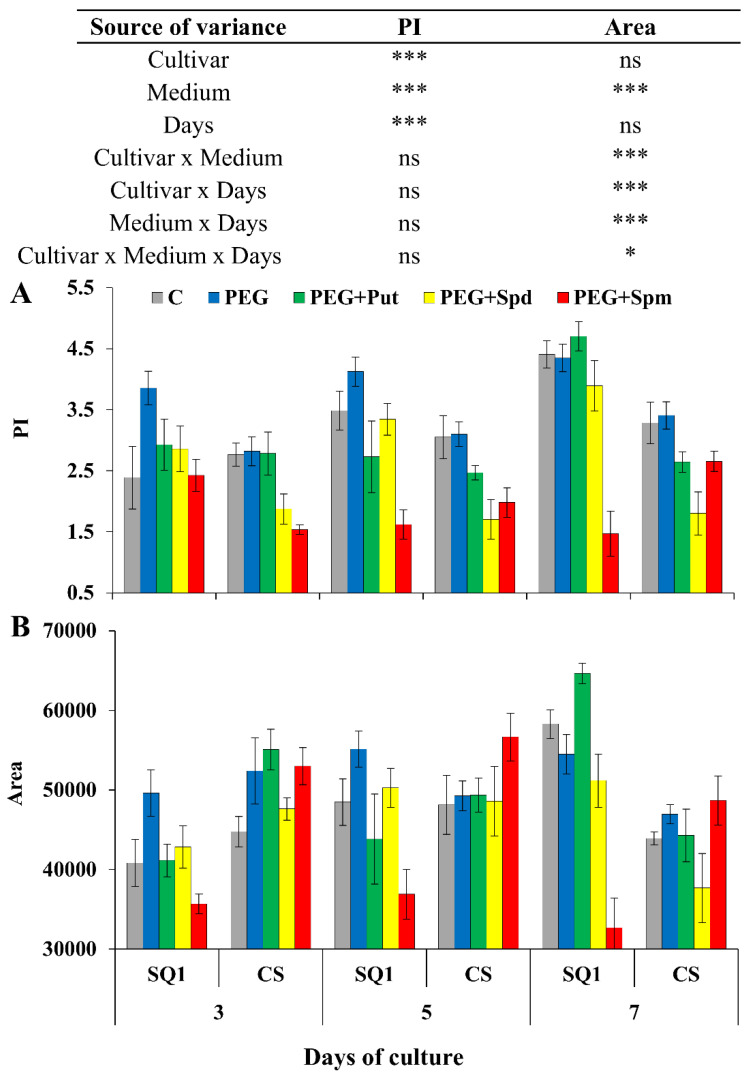
Chlorophyll *a* fluorescence parameters PI (**A)** and Area (**B**) in leaves of two wheat cultivars—CS and SQ1—on the 3rd, 5th and 7th day of PEG-induced osmotic stress and supplementation with PAs, resulting in a total of five treatments: Control, PEG, PEG + Put, PEG + Spd and PEG + Spm. The results of multiple factor analysis of variance (ANOVA) are presented in the table above the chart. The sources of variance of the measured parameters were as follows: two cultivars, five media, three measurement days, and their interactions. *, **, ***, significant at *p* ≤ 0.05, 0.01, 0.001, respectively; ns, not significant. Each bar represents a mean of 6 replicates ± SE.

Starting from the 3rd day of the treatment, for the majority of media and days, except for spermine supplementation, PI values significantly depended on the cultivar and were higher for SQ1 than for CS (Figure 3A). On the 3rd and 5th day, PEG caused a PI increase in both cultivars of about 38% and 8.5%, respectively, compared to control.

In the presence of spermine in the medium, in most cases the PI values were lower than in the presence of other PAs. In contrast, the influence of spermine on Area parameter changes was very high and those Area values were lower for SQ1 in comparison to CS on all measurement days (Figure 3B). On the 5th day, the presence of putrescine in the medium of SQ1 also resulted in a lower value than control, PEG and the presence of spermidine. Later, on the 7th day, putrescine caused the highest increase in Area for SQ1. On the 3rd and 5th day, Area values in the medium containing PEG were higher for SQ1 and CS compared to the control by ca. 15% and 8.5%, respectively. The presence of spermine caused a decrease in Area on the 3rd and 5th day for SQ1 in comparison to CS.

**Figure 4:**
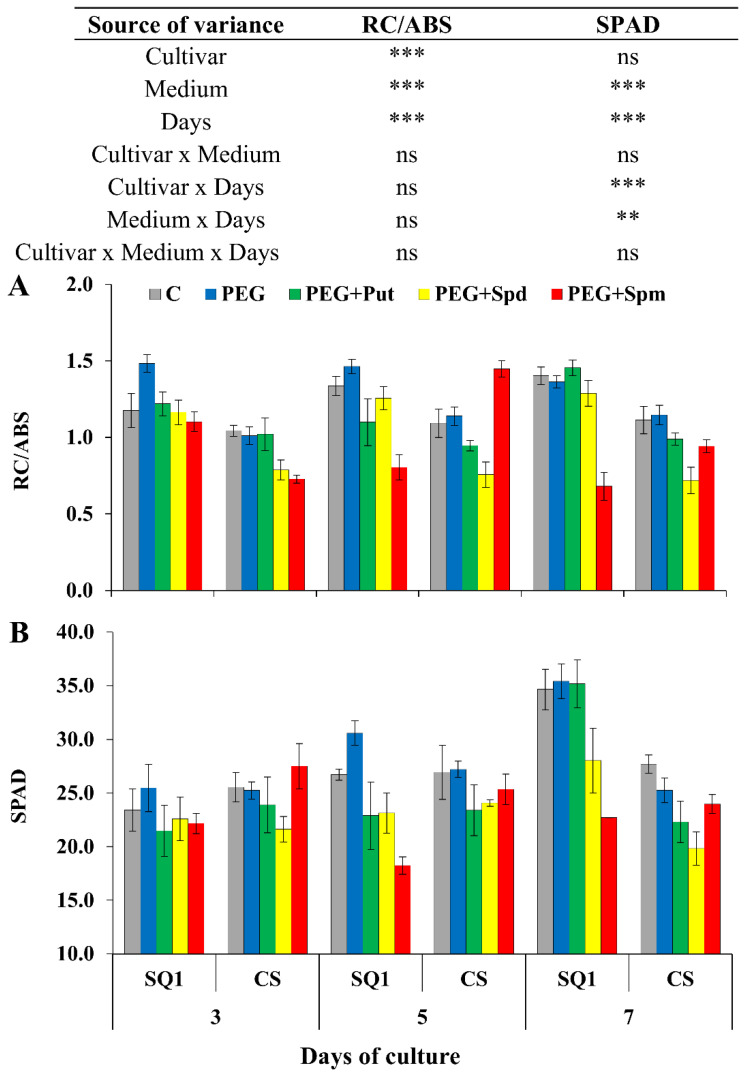
Chlorophyll *a* fluorescence parameter RC/ABS (**A**) and SPAD (**B**) in leaves of two wheat cultivars—CS and SQ1—on the 3rd, 5th and 7th day of PEG-induced osmotic stress and supplementation with PAs, resulting in a total of five treatments: Control, PEG, PEG + Put, PEG + Spd and PEG + Spm. The results of multiple factor analysis of variance (ANOVA) are presented in the table above the chart. The sources of variance of the measured parameters were as follows: two cultivars, five media, three measurement days, and their interactions. *, **, ***, significant at *p* ≤ 0.05, 0.01, 0.001, respectively; ns, not significant. Each bar represents a mean of 6 replicates ± SE.

Next, changes in the parameter associated with energy used for electron transport depending on the number of active reaction centers (RC/ABS) varied similarly to Area, and were significantly different for all sources of variance (cultivar, medium and day) (Figure 4A).

The presence of PEG in the medium did not have any influence on this parameter. On the 5th treatment day, the most visible effect on RC/ABS was observed in the presence of spermine for both cultivars, though it was an increase for CS and a decrease for SQ1. Exogenous application of PAs to the hydroponic medium caused significant changes in the chlorophyll content, measured in SPAD units. The pattern of changes was similar to those of the previously described parameter RC/ABS (Figure 4B). The effect of spermine was different from other PAs, control and PEG.

### 2.2. Biochemical Parameters

**Figure 5:**
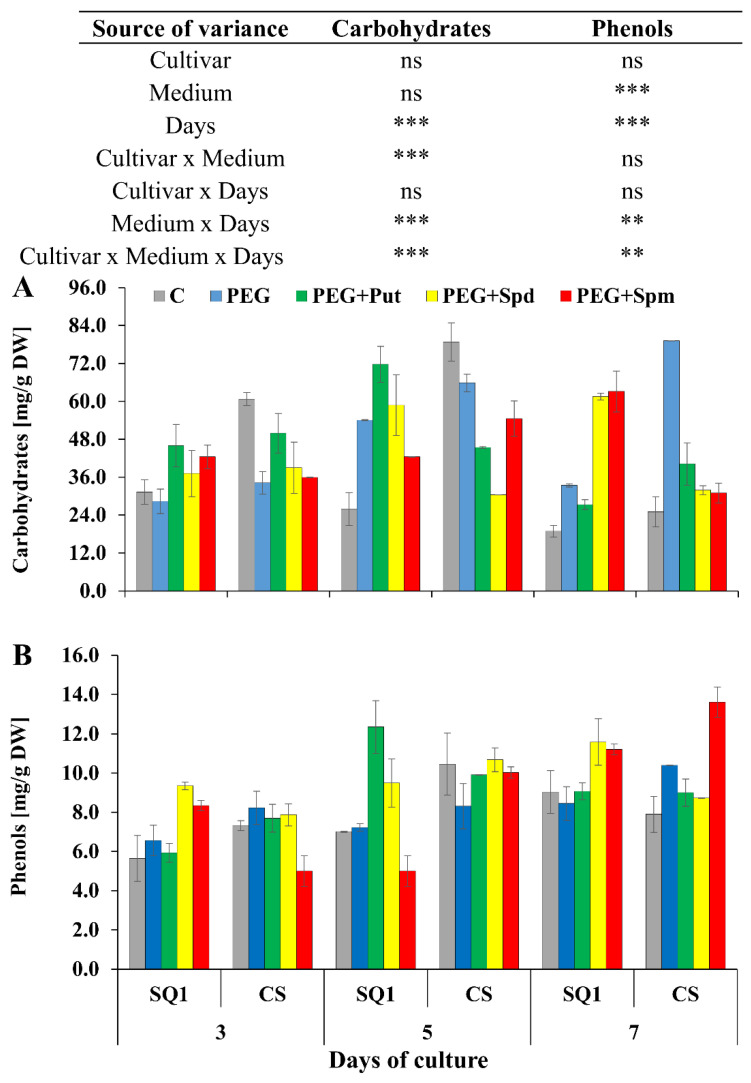
Carbohydrates (**A**) and phenols (**B**) in leaves of two wheat cultivars—CS and SQ1—on the 3rd, 5th and 7th day of PEG-induced osmotic stress and supplementation with PAs, resulting in a total of five treatments: Control, PEG, PEG + Put, PEG + Spd and PEG + Spm. The results of multiple factor analysis of variance (ANOVA) are presented in the table above the chart. The sources of variance of the measured parameters were as follows: two cultivars, five media, three measurement days, and their interactions. *, **, ***, significant at *p* ≤ 0.05, 0.01, 0.001, respectively; ns, not significant. Each bar represents a mean of 3 replicates ± SE.

During the growth of wheat seedlings in the control medium, the concentration of soluble carbohydrates on the 3rd and 5th day was higher for CS than for SQ1 and comparable (lower value) for both cultivars after 7 days of the treatment (Figure 5A). The presence of PEG in the medium caused a permanent increase in this parameter in CS, which lasted for all 7 days of the treatment.

In the other media this parameter was not significantly different on the 3rd and 5th day of the treatment. On the 7th day a difference between the cultivars was observed. Supplementation of the medium with spermidine and spermine caused a ca. two-fold increase in sugar content in SQ1 compared to CS. In the medium containing PEG, an over three-fold increase in carbohydrates was observed in CS compared to SQ1. In the case of phenols concentration such big differences were not noted while taking into account the cultivar and medium, especially supplementation with spermidine and putrescine (Figure 5B). Spermidine did not cause changes in the content of phenols in SQ1 compared to CS cultivar.

**Figure 6:**
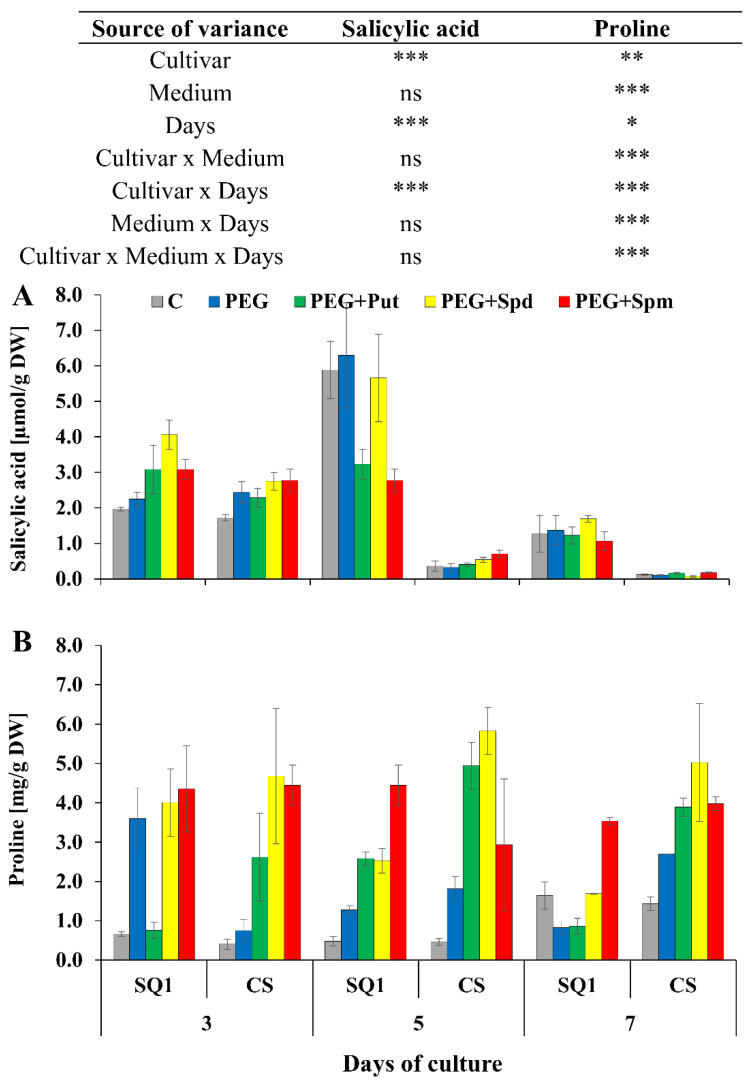
Salicylic acid (**A**) and proline (**B**) in leaves of two wheat cultivars—CS and SQ1—on the 3rd, 5th and 7th day of PEG-induced osmotic stress and supplementation with PAs, resulting in a total of five treatments: Control, PEG, PEG + Put, PEG + Spd and PEG + Spm. The results of multiple factor analysis of variance (ANOVA) are presented in the table above the chart. The sources of variance of the measured parameters were as follows: two cultivars, five media, three measurement days, and their interactions. *, **, ***, significant at *p* ≤ 0.05, 0.01, 0.001, respectively; ns, not significant. Each bar represents a mean of 3 replicates ± SE.

For SQ1, on the 5th day of the treatment, putrescine and spermidine caused the highest increase in phenols content—about 50% and 25%, respectively—compared to control and PEG medium. On the 7th day, supplementation with spermidine and spermine caused a ca. 20% increase in this parameter in SQ1, while supplementation with spermine resulted in a ca. 45% increase in CS. Another parameter measured on the 3rd, 5th and 7th day—salicylic acid content—was higher for the more susceptible SQ1 than for the more resistant CS. Already on the 3rd day, it appeared to be higher with PAs present in the medium compared to control and PEG. The increase was 28%, 50% and 28% in the presence of putrescine, spermidine and spermine, respectively (Figure 6A). After 5 and 7 days SA content dropped significantly in CS compared to SQ1. On the 7th day it decreased almost to 0 value, though it was independent of the kind of medium, including the type of supplemented PAs. Particularly interesting results were obtained for proline content in leaves of seedlings during 7 days of the treatment on media supplemented with PAs. While in control medium proline content was very low, it increased for both cultivars on the 7th day (Figure 6B). In contrast to the other parameters, an increase in proline content was observed for the more resistant CS compared to the susceptible SQ1. Supplementation of the medium with PAs caused an increase in proline content in this cultivar at all measurement points, especially when putrescine and spermidine were added to the medium. In the presence of spermine in the medium proline content remained practically at the same level for both cultivars.

**Figure 7:**
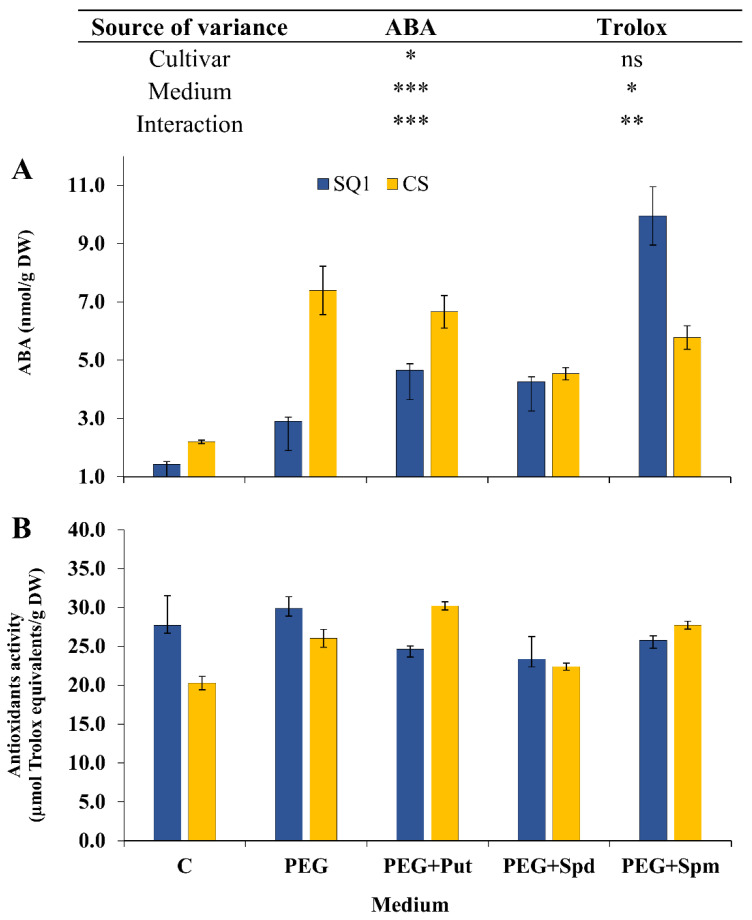
ABA level (**A**) and activity of low molecular weight antioxidants (**B**) in leaves of two wheat cultivars—CS and SQ1—on the 7th day of PEG-induced osmotic stress and supplementation with PAs, resulting in a total of five treatments: Control, PEG, PEG + Put, PEG + Spd and PEG + Spm. The results of two-way analysis of variance (ANOVA) are presented in the table above the chart. The sources of variance of the measured parameters were as follows: two cultivars, five media, and interaction between cultivar and medium. *, **, ***, significant at *p* ≤ 0.05, 0.01, 0.001, respectively; ns, not significant. Each bar represents a mean of 6 replicates ± SE.

ABA content in seedling leaves is generally considered as a good indicator of stress tolerance. In our experiment ABA content was measured only at the end of stress treatment, i.e., on the 7th day. It depended significantly on the cultivar, medium and their interaction (Figure 7A). In SQ1 osmotic stress generated by PEG treatment caused a two-fold increase in ABA content and a further increase was observed in the presence of putrescine and spermidine in the medium (3–4 fold) as well as in the presence of spermine (ca. 9-fold compared to control). In the more resistant CS, PEG treatment caused a substantial increase in ABA content (3.5-fold) compared to control. Medium supplementation with PAs maintained this increase—spermidine to the least extent.

The next parameter, i.e., the activity of low molecular weight antioxidants (measured as trolox equivalents) varied significantly depending on the medium but not on the cultivar (Figure 7B). PEG caused a ca. 12.5% increase in this parameter compared to control and for CS a further 12.5% increase was observed in the presence of putrescine. In the presence of spermidine and spermine the changes were negligible.

### 2.3. Water Content and Yield Components

When plants transferred from hydroponic culture to the soil reached maturity stage, we observed after-effects of PEG-induced osmotic stress combined with the supplementation of PAs (PEG, PEG + Put, PEG + Spd and PEG + Spm) on leaf water content (WC) and yield components such as the number and weight of grains (NG and WG) as well as biomass value per plant. It was observed that after PEG treatment WC and NG decreased for plants of both cultivars (Table 1). ANOVA analysis showed significant differences depending on the cultivar and medium only for WC and biomass. At that point, WC decreased by 38.4% for SQ1 and 20.8% for CS. At the same time, NG decreased by 3.4% for both cultivars. WG and biomass were at the same level for SQ1, whereas for CS biomass decreased by 10% compared to the control value. Supplementation of the medium with PAs caused a further decrease in WC—in particular, spermine induced a two-fold decrease in that parameter in both cultivars. In comparison to other PAs, spermine evened the yield components (NG, WG and biomass) in SQ1 to the control values (Table 1). Among all PAs, putrescine had the most striking effect on that cultivar, causing the greatest decrease in WC (55%) and yield components (NG, WG and biomass)—34.8, 36.9 and 37.4%, respectively—in comparison to control values. Other PAs—spermidine and spermine—had little effect on those parameters. For the more resistant CS, these effects were observed to a lesser extent. In this cultivar, the supplementation of the medium with putrescine decreased WC by 38% and biomass by 20% as well as slightly increased NG and WG. Spermidine caused a decrease of 39% in NG, 28% in WG and 20% in biomass, while the addition of spermine to the medium caused a decrease of 10% in NG, 14% in WG, and 21% in biomass. It proves that the addition of spermidine or spermine to the hydroponic medium was more beneficial for the more susceptible cultivar SQ1, keeping values of yield components closer to the control.

## 3. Discussion

Drought causes a number of changes in the course of physiological processes of plants, which result in modifications in numerous physiological parameters [23]. The plant, like any living organism, remains in a state of homeostasis, which is disturbed under the influence of drought. Thus, plant response to drought leads to a shifting of balance in the multidimensional network of physiological parameters. While counteracting or alleviating drought stress, the plant engages further mechanisms in order to restore natural balance. The hypothesis formulated in this work, postulating the alleviating effect of polyamines on these processes, required examining whether the application of polyamines results in restoring at least some of the measured parameters to the values observed in plants under optimal conditions, and distancing them from the values observed under the influence of PEG. However, we expect a different reaction if the studied parameter depends mainly on the intensity of repair mechanisms—then a significant discrepancy will be observed in the values obtained in non-supplemented plants compared to plants supplemented with polyamines, which activate the above-mentioned repair mechanisms.

Chlorophyll fluorescence is considered to be a useful tool for determining the effects of various stresses [24,25]. In the studies of Kalaji et al. [25], the photosynthetic efficiency of two barley cultivars was examined under the influence of different abiotic stresses with the use of the prompt fluorescence technique. The differences in the behaviour of the more and less susceptible cultivar were evaluated using chosen chlorophyll fluorescence parameters. The results indicated different mechanisms of tolerance and strategies for the conversion of light energy into chemical energy in the two cultivars, and confirmed the suitability of some chlorophyll fluorescence parameters as reliable biomarkers for screening plants at the level of photosynthetic apparatus efficiency. Stress can damage the PSII system, which leads to lower electron transfer rate and longer time required to reach maximum fluorescence intensity [26]. One of the aims of our study was to evaluate chlorophyll fluorescence as a criterion for the identification of stress resistant cultivars and the role of PAs in the alleviation of stress in controlled environments. It was shown that such parameters as F_v_/F_m_, F_v_/F_o_ of PSII during screenings, PI, Area, RC/ABS and the degree of greenness (chlorophyll content measured in SPAD units) could be treated as informative parameters for determining the physiological status of osmotic stress in wheat cultivars. F_v_/F_m_ and F_v_/F_o_ parameters differed significantly depending on the treatment day and the interaction between medium and day, though it was difficult to interpret which cultivar was more resistant to osmotic stress based on these parameters. In a large number of photosynthetic studies, the F_v_/F_m_ and F_v_/F_o_ ratios are used as stress indicators. However, these empirical parameters, based on F_o_ and F_m_ fluorescence values, are not always sensitive enough to detect differences between samples [24]. The next parameter—the performance index (PI)—depends on the reaction centre density and the electron transport efficiency. In our experiment, PI depended significantly on the cultivar—its value was higher for the more susceptible SQ1 than for the more resistant CS (Figure 3A). The presence of spermidine and putrescine in the medium practically equalized this parameter to the level of control, which shows that these polyamines ameliorate osmotic stress in the SQ1 cultivar. Throughout the treatment, spermine was ineffective in mitigating osmotic stress, which was manifested by a decrease in PI. The Area parameter refers to the area above the chlorophyll fluorescence curve between F_o_ and F_m_. It represents the pool of electron transporters in the electron transport chain [27]. In our experiment PEG caused an increase in this parameter in both cultivars compared to control during the first 5 days and in SQ1 a decrease on the 7th treatment day. Supplementation of the medium with spermine during 7 days of the treatment resulted in an increase in Area in the more resistant CS compared to control and the more susceptible SQ1. It is in accordance with the results of Kalai et al. [25], where after various stresses applied for 7 days, a significant increase in Area was observed in the more resistant barley cultivar in comparison to the control. ABS/RC represents the absorption flux per one active reaction centre (RC)—the ratio of active to inactive RCs [27]. The values of ABS/RC on the 5th and 7th treatment day in our study were higher for CS treated with PEG and also with spermine. Kalaji et al. [25] also reported that the more resistant barley cultivar had higher values of this parameter than the more sensitive one. Those results are in keeping with the data obtained for chlorophyll content, measured in SPAD units, where the CS cultivar was characterized by higher amount of chlorophyll.

Thus, in the case of the majority of the analysed chlorophyll fluorescence parameters, it can be assumed that they belong to those physiological parameters for which the effect of polyamines actually leads to the restoration of the state from before the occurrence of drought stress. This is quite understandable, as photosynthesis is a basic, essential physiological process and damage to its mechanisms inevitably leads to the death of the autotrophic organism that is the plant. Many other physiological processes occurring in the plant do not play such a fundamental role, and as such can be involved in restoring the homeostasis of basic processes. Below, we examine the impact of drought and polyamine application on the studied biochemical parameters in order to determine the nature and importance of these reactions.

The first of the studied parameters is the concentration of soluble sugars, which are important osmoprotectants. In the presented experiment, the application of polyamines caused an alleviation of stress effects, but only after a certain time had elapsed. This can be clearly seen in the more resistant CS cultivar on the 7th day of the treatment. Evidently, this process develops slowly, which is understandable, as carbohydrate accumulation requires the activation of many genes involved in different processes (CO_2_ assimilation, photosynthesis, Kalvin cycle, sugar processing) and the regulation of these processes [28]. Moreover, the synthesis of a greater amount of carbohydrates requires the use of an adequate amount of carbon compounds, while CO_2_ assimilation is difficult due to stomata closure. This conclusion is also supported by the results of the measurement of abscisic acid (ABA) level in both studied cultivars, which increased significantly in all PEG treatments. No similar effect of drought stress alleviation was observed in the cultivar susceptible to this stress. Therefore, the effects of drought stress are not only drastic and negative, but also long-lasting, with polyamines having no alleviating influence on them.

The measurement of ABA level also leads to the conclusion that the application of polyamines causes a decrease in the content of this hormone only in the resistant CS cultivar. In the susceptible SQ1 cultivar, polyamine application caused an increase in ABA accumulation in comparison to both control and plants treated only with PEG. ABA is a key compound in plant response to drought—its role is not only stomata closure and leaf abscission, but also stimulating the expression of many genes [29] controlled by the ABRE (ABscisic acid Response Element) sequence. Thus, a high ABA level indicates a high intensity of drought response processes, as the plant organism is still trying to avoid the lethal effects of drought and is reducing water loss through closed stomata, which results in reduced assimilation and lower content of the soluble sugars discussed earlier. All this indicates that treating the more susceptible cultivar with polyamines did not lead to an alleviation of drought effects. The exact opposite was observed for the drought resistant cultivar. The reduced accumulation of ABA due to polyamine application indicates a lower intensity of negative physiological effects of drought. The increased accumulation of soluble sugars in these plants points not only to efficient mechanisms of CO_2_ assimilation, but also to the intensification of the release of osmoprotectants that are soluble sugars. This reaction is far more beneficial for the plant than ABA accumulation, as it allows the plant to reduce water loss without inhibiting gas exchange (which is a side effect of stomata closure).

As a result of the application of polyamines, the activity of antioxidants in the susceptible cultivar slightly decreased in comparison to both control and PEG-treated plants (Figure 7B). The opposite effect was observed in the resistant cultivar, where these values increased slightly. Our explanation of this phenomenon is that the pool of antioxidants in resistant plants is not used up as much as in susceptible plants, where drought induces more intensive processes of reactive oxygen species production.

Proline is an indicator that correlates well with relative water content (RWC), as shown, e.g., by Zegaoui et al. [30]. In accordance with their studies, when we compared cultivars differing in drought tolerance, we observed higher proline levels in the cultivar more resistant to drought (Figure 6B). Thus, proline accumulation indicates not so much the occurrence of water stress in the plant, but rather the initiation of processes counteracting the effects of this stress. The observed changes in proline levels are consistent with this physiological role, with the drought susceptible SQ1 line initially accumulating large amounts of proline, which indicates the initiation of anti-stress processes, followed by a gradual decrease in proline levels over time. The drought resistant CS cultivar initially reacted poorly, but the response became stronger over time. The CS cultivar is characterized by a constant, elevated ABA level—also under optimal watering conditions—so these plants may not initially respond to drought due to the reduced transpiration rate caused by higher ABA levels [31]. In contrast, in the susceptible cultivar, drought stress caused a shock reaction, which led to serious physiological disturbances over time, including disruption and weakening of the mechanisms combating the effects of drought stress. The resistant cultivar activated its protective mechanisms slowly, adjusting the degree of their activation to the level of stress. The application of polyamines led to an increase in proline levels in the majority of treatments, which was particularly substantial in the resistant CS cultivar. Such a response indicates that polyamines do not work directly to alleviate the effects of drought stress, but instead activate protective mechanisms in the plant, with the degree of activation of these mechanisms depending on the genetic resources of the plant.

The stress-coping mechanisms include, among others, increased synthesis of antioxidants, which eliminate free radicals produced as a result of drought stress. The observed accumulation of antioxidants in plants of the resistant CS cultivar in response to stress, elevated even further after the application of polyamines, confirms the above-mentioned hypothesis.

Salicylic acid (SA) is another compound involved in regulating the response to drought stress. It enhances ABA-induced stomata closure [23]—its accumulation usually indicates a greater intensity of drought stress, but also leads to increased drought tolerance of the plant [32]. The observed differences in SA levels between cultivars and over the course of the experiment seem to point to a greater intensity of stress processes in plants of the cultivar less resistant to drought stress (SQ1), though it should be noted that ABA modulates the level of SA biosynthesis [33]. Thus, this effect may result from the inhibition of SA biosynthesis in the treatments characterized by high ABA levels, among other factors. The impact of polyamine application on SA accumulation was small, on the border of statistical error.

The dynamics of phenolics level changes were not significant, and it is also difficult to point to any considerable regularities. Interesting conclusions can be drawn when comparing SA (which is also a phenolic compound) level with phenolics accumulation, where the sharp decrease in SA level on the 5th day in CS and on the 7th day in SQ1 did not affect phenolics accumulation in any way. Therefore, it can be concluded that SA was inactivated (e.g., conjugated) or oxidized to polyhydroxyl derivatives, e.g., 2,3 dihydroxybenzoic acid or 2,5 dihydroxybenzoic acid—compounds which may have been formed in the reaction of SA with the hydroxyl radical [34].

In conclusion, the performed experiment revealed that the application of polyamines does not directly and unconditionally alleviate the effects of drought stress. Therefore, polyamines are not basic osmotically active compounds, like soluble sugars or proline. The level of endogenous polyamines is very small in comparison to other osmolyte [35], and their contribution to total osmotic potential is less than 1%. However, it is still possible that they can reach higher concentrations in organelles rich in DNA, proteins or other anionic compounds to which polyamines have affinity [36]. The other possible role of polyamines is signalling during drought stress. However, the effectiveness of polyamines in drought stress mitigation depends on the resources of a particular genotype—genotypes more tolerant to drought stress will trigger more effective mechanisms under the influence of polyamines. The physiological role of polyamines under drought stress remains unclear—while their role as a primary osmoprotectant seems questionable, their signalling function remains an open question, as the role of an easily available nitrogen source may be equally important [12,37], being crucial for the survival of the plant under drought stress.

## 4. Materials and Methods 

### 4.1. Plant Material 

Two hexaploid cultivars of wheat (*Triticum aestivum* L.)—Chinese Spring (CS) and SQ1—were examined in the study. The seeds of both studied genotypes were acquired from the John Innes Centre, Norwich, UK and then multiplied at our institute. The SQ1 cultivar, with increased content of ABA, was selected at the John Innes Centre from the seventh cross generation (F7) between two wheat cultivars: Highbury × TW269/9/3/4. CS and SQ1 differ significantly in their morphological traits—in comparison to CS, SQ1 is shorter with a smaller leaf surface area and fewer spikes with awns [31,38].

### 4.2. Growth Conditions

**Figure 8:**
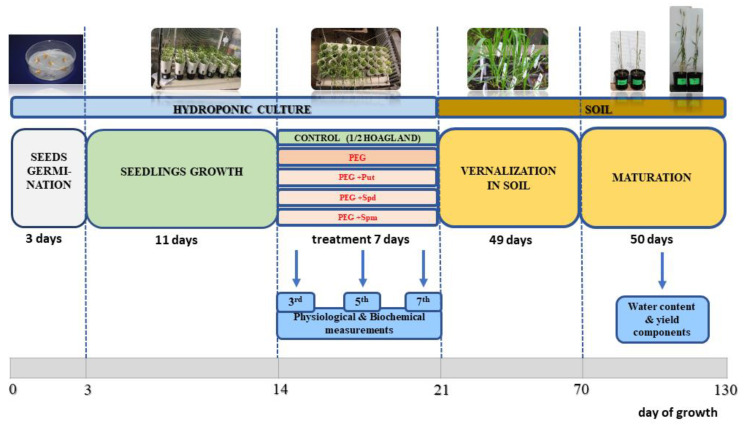
Scheme of the experimental set-up for hydroponically cultivated wheat seedlings treated with different exogenous polyamines under PEG-induced osmotic stress.

Preparation of grains was performed according to the procedure described in our previous papers [39,40]. In total, 330 germinating seedlings of CS and SQ1 cultivars were grown in 30 pots (11 plants per pot), each containing 30 mL half-strength Hoagland solution, and maintained in a hydroponic culture in a phytotronic greenhouse for 11 days. The hydroponic solution was aerated by air pumps. The pump flow was set to gentle aeration, the roots were immersed in the medium and the solution did not splash out, preventing a large loss of the culture medium. Every day, the hydroponic medium was supplemented with fresh medium, and every week it was completely exchanged with fresh medium. After 11 days of growth in control (C) conditions (25 °C, 16 h photoperiod, 400 μmol/m^2^ s light intensity), when the fourth leaf was fully expanded, seedlings were randomly divided into five groups: (1) C—control seedlings, grown only in half-strength Hoagland solution; (2) PEG—grown in C + PEG (−0.75 MPa); (3) PEG + Put; (4) PEG + Spd; and (5) PEG + Spm. PEG 6000 was used as it is frequently applied in physiological research to simulate osmotic stress. An 18% PEG solution was prepared to obtain PEG medium with an osmotic pressure of −0.75 MPa. PEG 6000 (180 g) was dissolved in 820 g of half-strength Hoagland’s medium and mixed on a magnetic stirrer with heating until PEG was completely dissolved. As regards PAs, 0.088 g of putrescine, 0.145 g of spermidine and 0.202 g of spermine were weighed and dissolved separately in 1 L of 18% PEG medium to obtain the necessary PA concentration. The pH of all solutions was adjusted to 6.0 and the seedlings were maintained in these media for the next 7 days. The concentration of polyamines was 1 mmol/L. At two-day intervals, the cultures were supplemented with 15 mL fresh solution. At the end of the treatment, i.e., after 7 days, one seedling from each pot (in three replicates) was transferred to the soil and placed in vernalization conditions (+4 °C, 8 h photoperiod) for 7 weeks. After that plants grew to maturity stage in optimal glasshouse conditions (25 °C, 16 h photoperiod). The scheme of the experiment is presented in Figure 8.

### 4.3. Physiological Parameters

On the 3rd, 5th and 7th day of PEG-induced osmotic stress and supplementation with PAs, selected parameters of chlorophyll *a* fluorescence were measured using a fluorometer (Handy PEA, Hansatech Instruments, King’s Lynn, UK). All measurements, performed in 6 replicates, were taken in the middle of the leaves, after darkening them with clips for a period of about 20 min. The following parameters were measured per fourth leaf cross-section: F_v_/F_m_ (maximal efficiency of PSII photochemistry), F_v_/F_o_ (maximum primary yield of PSII photochemistry), PI (performance index as an essential indicator of sample vitality), RC/ABS (reaction centers per antenna), Area (area over the chlorophyll *a* fluorescence induction curve; parameter informing about the number of acceptors available in PSII). Data were analyzed using a JIP test [25,27]. Chlorophyll content was measured in SPAD units using a hand-held meter (SPAD 502, Minolta Co. Ltd., Ōsaka, Japan).

### 4.4. Biochemical Parameters

Afterwards on the 3rd, 5th and 7th day of the treatment, the fourth leaf was excised as a sample and fixed in liquid nitrogen, frozen and stored at −70 °C. The samples were lyophilized and homogenized for the determination of biochemical parameters. The content of carbohydrates, phenols, proline and salicylic acid were evaluated. Additionally, at the end of PEG stress treatment and modifications with exogenous polyamines, water and abscisic acid content, as well as and the activity of low molecular weight antioxidants were measured.

#### 4.4.1. Soluble Carbohydrates

Spectrophotometric analysis of soluble sugar content was conducted according to Dubois et al. [41]. Carbohydrates were determined in ca. 5 mg of leaves extracted in 1.5 mL of 96% ethanol and centrifuged at 21,000 *g* for 5 min (Universal 32R, Hettich, Germany). The mixture containing 10 μL of the supernatant, 200 μL of distilled water, 200 μL of 5% phenol and 1 mL of 96% sulphuric acid was cooled and the absorbance was measured at 490 nm with a microplate reader (Synergy 2, BioTek, Winooski, VT, USA). The carbohydrates concentration was expressed in mg/g DW.

#### 4.4.2. Total Phenolics 

Total phenolics content was measured according to Singleton and Rossi [42]. Samples (50 mg of leaves) were homogenized in 1 mL of 80% (v/v) ethanol and centrifuged at 21,000 *g* for 5 min (Universal 32R, Hettich, Germany). Then, 20 μL of the supernatant was mixed with 500 μL of deionized water, 250 μL of 25% (w/v) Na_2_CO_3_ and 125 μL of Folin-Ciocalteu reagent. The absorbance was measured at 760 nm with a microplate reader (Synergy 2, BioTek, Winooski, VT, USA). The total phenolics content was expressed in mg/g DW.

#### 4.4.3. Proline 

Proline was determined according to modified Ting and Rouseff [43]. In short, samples of 5 mg of leaves were extracted with 0.5 mL of 3% 5-sulphosalicylic acid for 15 min and centrifuged at 39,000 *g* for 5 min (Mikro 22R, Hettich, Germany). The mixture of 200 μL of the supernatant, 200 μL of concentrated formic acid and 400 μL of 3% ninhydrin in 2-methoxyethanol was incubated for 0.5 h at 100 °C. The absorbance was measured at 514 nm with a microplate reader (Synergy 2, BioTek, Winooski, VT, USA). The proline concentration was expressed in mg/g DW.

#### 4.4.4. Salicylic Acid 

Salicylic acid (SA) was extracted with 1.5 mL of cold methanol from about 5 mg of leaves, and centrifuged at 38,000 *g* for 10 min (Universal 32R, Hettich, Germany) according to Wilbert et al. [44] modified by Dziurka et al. [45] The supernatant was processed with Oasis MCX SPE columns 1 cc/30 mg (Waters, Milford, MA, USA). SA content was analysed using the HPLC system Agilent 1260 equipped with the 6420 ESI Tandem Mass spectrometer and the SupelcoAscentis RP-Amide (7.5 cm × 4.6 mm, 2.7 μm) column. A gradient of 0.1% formic acid in water (A) and acetonitrile (B) at a flow rate of 0.5 mL/min was used. Two most abundant secondary ions (MRM) were used for the qualification and quantification of SA (primary ion 139.0, secondary ions 121.0 and 39.0) and D4SA (primary ion 143.1, secondary ions 125.1 and 41.1). The level of SA was expressed in µmol/g DW.

#### 4.4.5. Abscisic Acid

The content of abscisic acid (ABA) was measured in about 50 mg DW of leaves placed in 1.5 mL of cold distilled water, then kept in boiling water for 3 min, shaken overnight at 4 °C and centrifuged for 20 min at 18,000 *g* (MPW-350R, Warsaw, Poland). ABA was determined in the supernatant using indirect enzyme-linked immunosorbent assay (ELISA) according to Walker-Simmons and Abrams [46]. The antibody used was MAC 252 (Babraham Technix, Cambridge, UK). The absorbance was measured at 405 nm with a spectrophotometer (Model 680, Bio-Rad Laboratories, Inc., Hercules, CA, USA). For each treatment, at least six independent ELISA measurements were made on three independent samples each collected from three different plants.

#### 4.4.6. Total Low Molecular Weight Antioxidant Activity

For the measurement of total antioxidant activity, 30 mg DW g of leaves was homogenized with 1.5 mL of 50% ethanol, shaken for 2 h at room temperature and then centrifuged for 20 min at 18,000 *g* (MPW-350R, Warsaw, Poland). The total activity of low molecular weight antioxidants was determined by 1,1-diphenyl-2-picrylhydrazyl (DPPH) method according to Brand-Williams et al. [47] with some modifications adapting the protocol to 96-well microtiter plates and to the measurement of absorbance with a microtiter plate reader [48]. The absorbance was measured at 490 nm with a spectrophotometer (Model 680, Bio-Rad Laboratories, Hercules, CA, USA). For each treatment at least six measurements were made on three independent samples each collected from two different plants. The antioxidant activity was expressed as μmoles of Trolox equivalents.

### 4.5. Water Content and Yield Components

At the end of the treatment, i.e., after 7 days, one seedling from each pot (in three replicates) was transferred to the soil and placed in the vernalization conditions (+4 °C, 8 h photoperiod) for 7 weeks. After that plants grew to maturity stage in optimal glasshouse conditions (25 °C, 16 h photoperiod) in order to observe the aftereffects of the prior stress treatment on the water content and yield characteristics (number and weight of grains, as well as biomass value per plant) were evaluated. Water content in leaves was determined as H_2_O content in grams using the formula: (FW-DW)/DW, where FW is the fresh weight and DW is the dry weight of leaves [49].

### 4.6. Statistical Analysis

The experiment was performed in a completely randomized design. The results presented in the figures are mean values ± standard error (SE) based on three (carbohydrates, phenolics, **s**alicylic acid, proline, water content and yield components) or six (fluorescence chlorophyll *a* parameters, SPAD, ABA content and total low molecular weight antioxidant activity) replicates. The statistical analysis of the data for each parameter was conducted using multivariate analysis of variance (ANOVA) with the statistical package Statistica 13.1 (StatSoft, Tulsa, OK, USA).

## Figures and Tables

**Table 1:** Leaf water content (g H_2_O g^−1^ DW), yield components: number of grains per plant, grain weight (g) and biomass (g) per plant in Chinese Spring (CS) and SQ1 wheat cultivars depending on the treatment: Control, PEG, PEG + Put, PEG + Spd and PEG + Spm. The results of two-way analysis of variance (ANOVA) are presented below the table. The sources of variance of the measured parameters were as follows: two cultivars, five media, and interaction between cultivar and medium. *, **, ***, significant at *p* ≤ 0.05, 0.01, 0.001, respectively; ns, not significant. Mean values ± SE, number of replicates = 6 (WC), 3 (NG, WG, Biomass).

Cultivar	Medium	Water Content (WC)	Number of Grains (NG)	Weight of Grains (WG)	Biomass
SQ1	Control	7.5 ± 0.2	82.3 ± 9.9	3.5 ± 0.5	6.0 ± 0.8
	PEG	4.7 ± 0.4	79.6 ± 4.6	3.5 ± 0.2	6.0 ± 0.4
	PEG + Put	3.4 ± 0.2	60.6 ± 5.3	2.3 ± 0.1	4.1 ± 0.1
	PEG + Spd	3.3 ± 0.6	82.6 ± 4.7	3.4 ± 0.2	5.3 ± 0.4
	PEG + Spm	3.8 ± 0.3	89.3 ± 5.4	3.5 ± 0.7	6.0 ± 1.0
CS	Control	5.3 ± 0.3	222.6 ± 21.9	5.7 ± 0.3	13.2 ± 1.0
	PEG	4.2 ± 0.2	215.0 ± 5.20	5.8 ± 0.5	11.6 ± 0.8
	PEG + Put	3.3 ± 0.2	229.3 ± 18.8	5.9 ± 0.5	10.8 ± 1.4
	PEG + Spd	3.2 ± 0.3	159.0 ± 24.9	4.1 ± 0.4	8.7 ± 1.0
	PEG + Spm	2.2 ± 0.4	180.6 ± 10.9	4.9 ± 0.1	10.5 ± 0.0
**Source of Variance**		**WC**	**NG**	**WG**	**Biomass**
Cultivar		**	***	***	***
Medium		***	ns	ns	*
Cultivar × Medium		ns	*	*	ns

## Abbreviations

ABA	abscisic acid
Area	area over the chlorophyll *a* fluorescence induction curve
C	control
CF	chlorophyll *a* fluorescence
CS	Chinese Spring
DW	dry weight
F_v_/F_m_	maximal efficiency of PSII photochemistry
F_v_/F_o_	maximum primary yield of PSII photochemistry
FW	fresh weight
NG	number of grains
Pas	polyamines
PEG	polyethylene glycol
PI	performance index as an essential indicator of sample vitality
Put	putrescine
RC/ABS	reaction centers per antenna
SA	salicylic acid
SPAD	chlorophyll content units
Spd	spermidine
Spm	spermine
SQ1	wheat cultivar
WC	water content
WG	weight of grains
